# *Bacteroides* *finegoldii* and *Parabacteroides goldsteinii* Mediate Fucoidan-Induced Attenuation of Intestinal Inflammation in Mice Through Betaine- and Spermidine-Related Pathways

**DOI:** 10.3390/foods15020203

**Published:** 2026-01-07

**Authors:** Tao Qin, Yifan Wei, Weiyun Zheng, Shugang Li, Shuang Song, Chunqing Ai

**Affiliations:** 1 The Third Clinical School of Medical Science, Qingdao University, Qingdao Municipal Hospital, Qingdao 266071, China; qintao@qdsslyy.wecom.work; 2 School of Food Science and Technology; National Engineering Research Center of Seafood, Dalian Polytechnic University, Dalian 116034, China; 13233082911@163.com (Y.W.); lishugang688@163.com (S.L.); songs1008@163.com (S.S.); 3 School of Agronomy and Life Science, Shanxi Datong University, Datong 037009, China; zhengweiyun0607@163.com

**Keywords:** *Bacteroides*, *Parabacteroides*, intestinal inflammation, metabolites

## Abstract

Fucoidan improves host health by enriching beneficial taxa such as *Bacteroides* and *Parabacteroides*, yet the underlying mechanisms remain unclear. This study validated the association between these two genera and fucoidan-mediated mitigation of intestinal inflammation in mice. Subsequently, the effects of *Parabacteroides goldsteinii* and *Bacteroides finegoldii* were evaluated in colitis mice, and the contributions of microbiota-associated metabolites spermidine and betaine were investigated in vitro. Both strains reduced IL-6 (32–36%), TNF-α (30–37%), and IL-1β (40–45%) levels and increased levels of catalase (25–35%) and glutathione peroxidase (31–45%) in the colon. Mechanically, these strains suppressed activation of the NF-κB and MAPK pathways and preserved tight junction integrity by inhibiting myosin light chain kinase activation. These effects were associated with alterations of gut microbiota, characterized by decreased Proteobacteria and increased Bacteroidota, resulting in increased betaine (45–60%) and spermidine (90–112%). In vitro, betaine and spermidine alleviated LPS-induced inflammation and oxidative damage by regulating macrophage polarization. These results suggest that *Bacteroides* and *Parabacteroides* contribute to fucoidan-induced improvement of host health through betaine- and spermidine-related pathways. Future studies should clarify the origins of key metabolites and validate their causality and translational relevance using approaches such as fecal microbiota transplantation, metabolite tracing, and human-relevant systems.

## 1. Introduction

Inflammatory bowel diseases (IBD), comprising Crohn’s disease and ulcerative colitis, are chronic immune-mediated disorders. Their incidence shows marked geographical variation, with the highest rates in developed countries and rapidly increasing trends in many developing regions [1]. The etiology of IBD is multifactorial, involving environmental factors, disruption of the intestinal barrier, genetics, and immune dysfunction [2]. Although current therapies, including 5-acetylsalicylic acid, corticosteroids, monoclonal antibodies, and biologics, alleviate IBD symptoms, they often require long-term administration and are associated with resistance or adverse effects [3,4]. When pharmacological options fail, surgical intervention is required, highlighting the urgent need for novel therapeutic approaches or complementary strategies to enhance existing treatments.

Emerging evidence supports the therapeutic potential of dietary compounds in promoting intestinal homeostasis and managing IBD [5]. For instance, polysaccharides exert notable immunomodulatory activities and are considered promising candidates for IBD therapy [6]. Probiotics, prebiotics, and synbiotics have demonstrated efficacy in the control of acute exacerbations and maintenance of remission [7]. Bioactive peptides also alleviate intestinal disorders by protecting against pathogenic infections and suppressing inflammatory responses [8]. Moreover, small molecules such as flavonoids, phenolic acids, and fatty acids exhibit anti-inflammatory effects in various IBD models [9,10]. Although the mechanisms underlying these benefits are not yet fully elucidated, modulation of gut microbiota has been recognized as a central mechanism.

Gut microbiota plays a central role in maintaining host homeostasis, and its dysfunction is linked to intestinal diseases, largely through disruption of the epithelial barrier and activation of inflammatory pathways [11]. Polysaccharides have been widely reported to ameliorate IBD by strengthening barrier integrity and reshaping microbial communities. During such interventions, beneficial taxa such as *Lactobacillus*, *Bacteroides*, *Bifidobacterium*, and *Parabacteroides* are frequently enriched and are thought to contribute to the therapeutic effects. In particular, *Parabacteroides* and *Bacteroides* species respond strongly to various polysaccharides due to their capacity to metabolize complex polysaccharides [12]. Notable strains, including *Bacteroides vulgatus* [13], *Parabacteroides distasonis* [14], and *Bacteroides uniformis* [15], have demonstrated anti-inflammatory potential in mouse models. However, the association between specific taxa and polysaccharide-mediated benefits remains to be fully validated, and their beneficial effects and underlying mechanisms require further clarification.

In this study, the association between *Bacteroides*/*Parabacteroides* and fucoidan-mediated alleviation of intestinal inflammation was validated in mice challenged by *Salmonella*. Subsequently, the effects of fucoidan-responsive strains, *B. finegoldii* and *P. goldsteinii*, on colonic inflammation were evaluated in mice by measuring histological injury and inflammatory responses. Mechanistically, their effects on the NF-κB and MAPK pathways, barrier integrity, myosin light chain kinase (MLCK), and oxidative stress were assessed. In addition, alterations in the gut microbiota and metabolites were examined via microbiome and metabolomic analysis. Finally, the effects of key metabolites, betaine and spermidine, on inflammatory response were validated in in vitro models. This study supports the role of *Bacteroides* and *Parabacteroides* as key functional mediators of fucoidan-derived intestinal protection.

## 2. Materials and Methods

### 2.1. Materials and Reagents

Fucoidan was prepared as previously described [16]. Briefly, dried *Undaria pinnatifida* powder was mixed with ethanol overnight at a 1:5 (*w*/*v*) ratio, followed by centrifugation at 5000 rpm for 5 min. The resulting residue was suspended in water at a 1:10 ratio (*w*/*v*) and incubated at 50 °C for 2 h, after cellulase and pectinase were added. Following centrifugation, the supernatant was precipitated with ethanol to a final concentration of 30% (*v*/*v*) to remove alginate. Ethanol was subsequently added to the remaining solution to a final concentration of 70% (*v*/*v*). The resulting precipitate was collected and further purified by two additional precipitations with 70% ethanol. The final precipitate was designated as fucoidan. Cefradine (C804452), gentamicin sulfate (G6064), and metronidazole (M815097) were obtained from Macklin Biochemical Technology Co., Ltd. (Shanghai, China). RIPA lysis buffer, phenylmethylsulphonyl fluoride (PMSF), and BeyoECL kit were purchased from Biyuntian (Shanghai, China). Citrate and Tris-EDTA antigen retrieval buffer were obtained from Servicebio (Wuhan, Hubei, China). PVDF membrane was purchased from Millipore (Burlington, MA, USA). Catalase (CAT), myeloperoxidase (MPO), total antioxidant capacity (T-AOC), inducible nitric oxide synthase (iNOS), malondialdehyde (MDA), superoxide dismutase (SOD), and glutathione peroxidase (GSH-PX) assay kits were obtained from Jiancheng Bioengineering Institute (Nanjing, Jiangsu, China). Nitric oxide (NO) assay kit was obtained from Beyotime (Shanghai, China).

### 2.2. Bacteria Culture

*Salmonella enterica* serovar Typhimurium ATCC14028 (ST) was cultured in Luria–Bertani broth at 37 °C, collected, and washed twice with PBS. The bacterial pellet was resuspended in PBS at 5 × 10^5^ CFU/mL. *B. finegoldii* (BF, GeneBank: MK743931) and *P. goldsteinii* (PG, Genebank: MK696407), isolated and stored in our laboratory [17], were cultured in Brain Heart Infusion medium and resuspended in PBS to 5 × 10^9^ CFU/mL.

### 2.3. Animal Experiments

#### 2.3.1. Ethics Statement

Male C57/BL6 mice (specific pathogen-free, 8 weeks old) were obtained from Changsheng Biotechnology Co., Ltd. (Benxi, Liaoning, China) and kept at the animal facility under standard conditions (23–25 °C, 45–55% humidity, 12 h light/dark cycle). All procedures were conducted in accordance with the guidelines of the National Institutes of Health and were approved by the Animal Ethics Committee of Dalian Polytechnic University (DLPU2021051; approval date: October 2021). Blood was collected from the orbital sinus under isoflurane anesthesia, followed by cervical dislocation.

#### 2.3.2. Mouse Model-1

Mice were divided into control (Con), ST-infected (Mod), and fucoidan-treated (Fuc) groups, *n* = 7/group. The Mod and Fuc groups received 200 μL of antibiotic cocktail (gentamicin sulfate (23 mg/kg), cefradine (23 mg/kg), and metronidazole (30 μg/kg)) by oral gavage daily for 10 days [18], followed by 200 μL of ST suspension for 13 days. During the infection phase, the Fuc group was additionally treated with fucoidan (200 mg/kg/day), while the Con group was given PBS. After euthanasia, the liver, colon, and spleen were collected, and the organ index (organ weight/body weight ratio) was calculated as previously described [19].

#### 2.3.3. Mouse Model-2

Mice were divided into four groups (*n* = 7/group): control (NC), DSS-treated (DSS), *B. finegoldii*-treated (BF), and *P. goldsteinii*-treated (PG). The DSS, BF, and PG groups received 4% (*w*/*v*) DSS solution (freshly prepared and replaced daily) for 7 days, whereas the NC group received water. BF and PG mice were additionally gavaged daily with 200 μL of corresponding bacterial suspension, while the NC and DSS groups received 200 μL PBS. Body weight was recorded daily.

### 2.4. Diseases Activity Index (DAI)

The severity of colonic inflammation was evaluated using the DAI as previously described [20].

### 2.5. Biochemical Assays

Colon tissues were homogenized in PBS with a glass homogenizer at a ratio of 1:9 (*w*/*v*) and centrifuged at 1000 rpm, 4 °C for 10 min to collect supernatants. Cytokine levels in supernatants were quantified using commercial kits (Jiancheng Bioengineering Institute; Nanjing, Jiangsu, China). Levels of CAT, GSH-PX, T-AOC, T-SOD, MPO, MDA, and iNOS in supernatants or cell samples were determined using kits. Absorbance was recorded using a microplate reader at the recommended wavelengths, and all assays were performed in duplicate.

### 2.6. Histological Analysis

Distal colon tissues were fixed, dehydrated, cleared, and embedded in paraffin. The blocks were sectioned at a thickness of 5 μm, deparaffinized, rehydrated, and stained with hematoxylin and eosin (H&E) to evaluate histopathological changes, including inflammatory cell infiltration, epithelial loss, and crypt destruction [21]. For assessment of mucosal barrier and goblet cells, adjacent sections were stained with Alcian blue (AB) and periodic acid Schiff (PAS) according to standard protocols to visualize acidic and neutral mucin [22]. Histological images were captured using a light microscope, and pathological evaluation was performed in a blinded manner.

### 2.7. Western Blotting

Protein samples were separated using SDS-PAGE and transferred onto PVDF membranes. Membranes were blocked for 1 h, washed, and incubated overnight at 4 °C with diluted primary antibodies: anti-TLR4 (38519), p65 (8242T), p-IκBα (2859T), p-p38 (8690), p-p65 (3033T), p38 (4511), p-MLC2 (3671T), ZO-1 (8193), and claudin-1 (4933) (CST, Danvers, MA, USA), and anti-occludin (ab216327), MLCK (ab232949), MLC2 (ab92721), IκBα (ab76429), JNKs (ab179462), p-JNKs (ab124956), ERKs (ab184699), and p-ERKs (ab201015) (Abcam, Cambridge, UK). After washing, membranes were incubated with a secondary antibody. Protein bands were visualized using chemiluminescent reagent and imaged with a ChemiDoc Touch system (Hercules, CA, USA). Band intensities were quantified using ImageJ software (1.5.3).

### 2.8. Immunohistochemistry

Immunohistochemical staining of MUC2 and tight junction proteins was conducted by Servicebio (Wuhan, Hubei, China) [18]. Briefly, sections were deparaffinized, retrieved, and blocked with H_2_O_2_ and rabbit serum. Then, sections were incubated with diluted primary antibodies, followed by a secondary antibody. The sections were counterstained with diaminobenzidine and hematoxylin, and the expression of targeted proteins was visualized using optical microscopy.

### 2.9. Analysis of the Microbiota Composition

Fecal microbiota composition was analyzed using 16S rRNA gene sequencing (v3–v4 regions) at Novogene Bioinformatics Technology Co., Ltd. (Beijing, China) [23]. Microbial richness and diversity were assessed using Chao1 and Shannon indices. Principal coordinate analysis (PCoA) was performed to evaluate inter-group similarities. Differentially abundant taxa were identified using linear discriminant analysis (LDA) effect size.

### 2.10. Analysis of Microbiota Metabolite Profile

Microbiota metabolites were analyzed as described in our previous study [24]. Inter-group differences were assessed through multivariate analysis, including principal component analysis (PCA) and orthogonal partial least squares-discriminant analysis (OPLS-DA). Differential metabolites were visualized using volcano plots, and potential metabolic pathways were annotated via KEGG enrichment analysis.

### 2.11. In Vitro Cell Experiments

#### 2.11.1. Cell Culture

RAW264.7 cells were cultured in DMEM medium with 10% fetal bovine serum, penicillin (100 U/mL), and streptomycin (100 μg/mL) at 37 °C in a humidified incubator with 5% CO_2_.

#### 2.11.2. MTT and NO Assays

RAW264.7 cells were seeded into 96-well plates at 2 × 10^4^ cells/mL (100 µL/well) and incubated for 24 h. Cells were treated with various concentrations of spermidine (Spd, 0–50 μmol/L) or betaine (Bet, 0–400 μmol/L). NO production was quantified using the Griess reagent method [25]. Cell viability in the MTT assay was measured at 570 nm [26].

#### 2.11.3. RT-PCR

Total RNA was extracted using TRIzol reagent, and cDNA synthesis was performed with PrimeScript™ RT reagent kit (Takara; Dalian, China). Gene expression was quantified in a qTower RT-PCR system with specific primers (Table S1). Relative mRNA expression was normalized to β-actin and calculated using the 2^–∆∆Ct^ method [27].

#### 2.11.4. Intracellular Reactive Oxygen Species (ROS) Detection and Hoechst Staining

Intracellular ROS were assessed using DCFH-DA staining as previously described [28]. Nuclear morphology was evaluated by Hoechst staining [29].

#### 2.11.5. Immunofluorescence

Cells were fixed, permeabilized, and blocked with 5% BSA. After incubation with primary antibodies (CD86 and CD163), cells were washed and incubated with a secondary antibody for 1 h. Nuclei were counterstained with DAPI, followed by examination using a fluorescence microscope.

### 2.12. Statistical Analysis

Data are presented as mean ± SD. Statistical analysis was performed using GraphPad Prism 8.0 (GraphPad software; San Diego, CA, USA). Homogeneity of variances was assessed using Bartlett’s test. One-way analysis of variance followed by Tukey’s multiple comparison tests was used for comparisons among groups. *p* < 0.05 was considered statistically significant. * *p* < 0.05, ** *p* < 0.01, and *** *p* < 0.001.

## 3. Results and Discussion

### 3.1. Bacteroides and Parabacteroides Correlated with Fucoidan-Mediated Attenuation of Intestinal Inflammation

**Figure 1:**
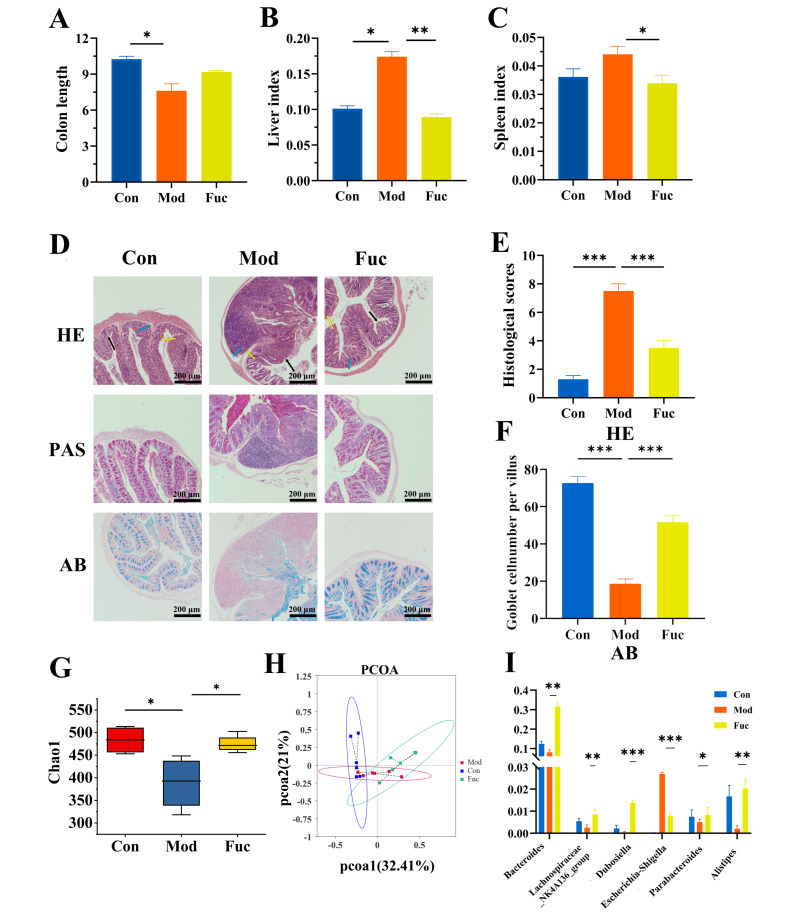
Fucoidan ameliorated ST-induced intestinal inflammation. (**A**) Colon length. (**B**) Liver index. (**C**) Spleen index. (**D**) Histological analysis based on HE, PAS, and AB staining. Arrows indicate inflammatory infiltration (yellow), crypt architecture (black), and epithelial destruction (blue); scale bar = 200 μm. (**E**) Histological scores. (**F**) Goblet cell number per villus. (**G**) Chao1 index. (**H**) PCoA. (**I**) Relative abundances of specific genera. * *p* < 0.05, ** *p* < 0.01, and *** *p* < 0.001.

Fucoidan has been reported to promote host health by enriching beneficial gut genera, particularly *Bacteroides* and *Parabacteroides*, across diverse disease contexts. For example, fucoidan alleviated IBD in mice in parallel with increased *Bacteroides* abundance [30] and improved metabolic disorders accompanied by enrichment of *Parabacteroides* [31]. To further examine the association of these genera with intestinal inflammation, we evaluated the effect of fucoidan in a mouse model of ST-induced intestinal injury. ST challenge caused significant colonic shortening and increased liver and spleen indices (Figure 1A–C), whereas fucoidan markedly normalized these changes. Fucoidan reduced inflammatory cell infiltration, crypt loss, and epithelial disruption, resulting in an improved histological score (Figure 1D,E). AB/PAS staining revealed a reduction in mucin and goblet cells in Mod mice (Figure 1F), which was restored by fucoidan.

Furthermore, gut microbiota analysis showed fucoidan increased microbial richness (Chao1 index, Figure 1G) and reshaped the overall community structure (Figure 1H). Notably, fucoidan enriched several taxa, including Lachnospiraceae NK4A136, *Bacteroides*, *Parabacteroides*, *Alistipes*, and *Dubosiella*, while reducing *Escherichia*-*Shigella* levels (Figure 1I). These results suggest that *Parabacteroides* and *Bacteroides* are associated with fucoidan-mediated protection against intestinal inflammation. Similar microbiota signatures have also been reported for other polysaccharides, such as those from *Enteromorpha clathrate* [32] and *Agrocybe cylindracea* [33]. These results identify these two genera as potential microbial mediators of fucoidan-driven anti-inflammatory effects and highlight their mechanistic relevance in polysaccharide-associated intestinal protection. This is consistent with current perspective that these genera represent promising next-generation probiotic candidates for targeted disease management [34].

### 3.2. P. goldsteinii and B. finegoldii Alleviated Colitis and Systemic Organ Injury

**Figure 2:**
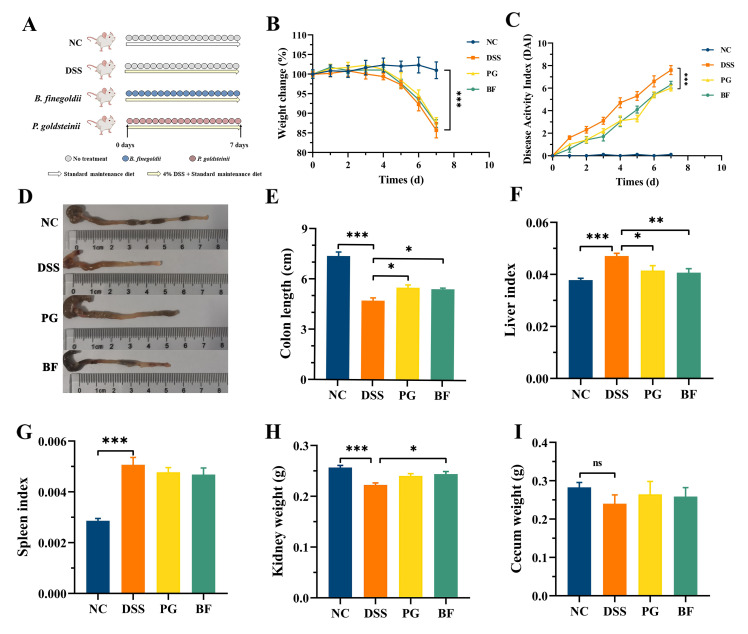
PG and BF ameliorated colitis. (**A**) Experimental process. (**B**) Body weight. (**C**) DAI scores. (**D**) Colon morphology (**E**) and length. (**F**) Liver index. (**G**) Spleen index. (**H**) Kidney weight. (**I**) Cecum weight. * *p* < 0.05, ** *p* < 0.01, and *** *p* < 0.001. ns = not significant.

Given their ability to metabolize fucoidan [35], BF and PG were selected for evaluation in a DSS-induced colitis model. DSS-treated mice showed marked weight loss, elevated DAI scores, and shortened colon length (Figure 2B–E). Although neither PG nor BF prevented weight loss, both strains markedly reduced DAI scores and partially restored colon length. DSS treatment also increased liver and spleen indices and reduced kidney weight (Figure 2F–H). BF and PG markedly reduced liver index, whereas only BF increased kidney weight. Cecum weight did not differ significantly among groups (Figure 2I). No significant differences were observed between BF and PG for these parameters. The protective effects are consistent with previous reports on *B. vulgatus* Bv46 [13] and *P. distasonis* [36].

### 3.3. P. goldsteinii and B. finegoldii Mitigated Inflammation by Inhibiting the NF-κB Pathway

**Figure 3:**
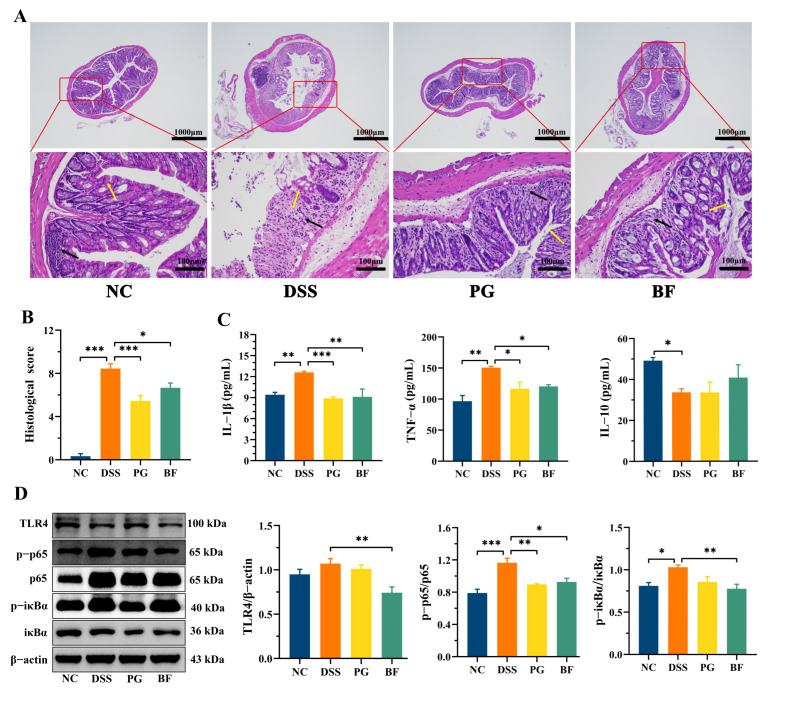
PG and BF mitigated inflammation by suppressing the TLR4/NF-κB signaling. (**A**) Representative HE-stained images of colon tissues (upper, scale bar = 1000 μm) and (lower, scale bar = 100 μm). Arrows indicate inflammatory infiltration (black) and crypt architecture disruption (yellow). (**B**) Histological scores. (**C**) TNF-α, IL-1β, and IL-10 levels. (**D**) Western blot analysis and quantification of NF-κB pathway-related proteins. * *p* < 0.05, ** *p* < 0.01, and *** *p* < 0.001.

DSS-treated mice exhibited typical colitis-associated pathological features, including crypt loss, barrier disruption, inflammatory infiltration, and submucosal edema, resulting in significantly elevated histological scores (Figure 3A,B). PG and BF treatment markedly mitigated these pathological changes and reduced histological scores. Pro-inflammatory cytokine IL-1β and TNF-α levels were markedly elevated in DSS-treated mice, whereas IL-10 was reduced. BF and PG markedly reduced IL-1β and TNF-α in DSS-treated mice, while BF showed a modest increase in IL-10 (*p* > 0.05, Figure 3C). To elucidate the underlying mechanisms, activation of the TLR4/NF-κB pathway was examined. TLR4 recognizes microbial molecules such as LPS and triggers the NF-κB signaling via phosphorylation of p65 and IκBα, leading to pro-inflammatory cytokine production [37]. DSS treatment resulted in elevated TLR4 expression (*p* > 0.05) and markedly increased p-p65/p65 and p-IκBα/IκBα (Figure 3D), indicating enhanced NF-κB activation. BF treatment markedly downregulated TLR4 expression and reduced p-p65/p65 and p-IκBα/IκBα, while PG reduced p-p65/p65 without significantly affecting TLR4 expression and p-IκBα/IκBα. These results suggest that both strains mitigate intestinal inflammation through modulation of the TLR4/NF-κB pathway. Similar inhibitory effects on the TLR4/NF-κB pathway have been reported for *P. distasonis* ATCC8503 and *B. thetaiotaomicron* DSM2079 [38,39]. However, contrasting reports indicate strain-dependent pro-inflammatory effects of some *Bacteroides* species [40], underscoring that the immunomodulatory roles of *Bacteroides* and *Parabacteroides* are highly strain- and context-dependent.

### 3.4. P. goldsteinii and B. finegoldii Attenuated Oxidative Stress in Colon Tissues

**Figure 4:**
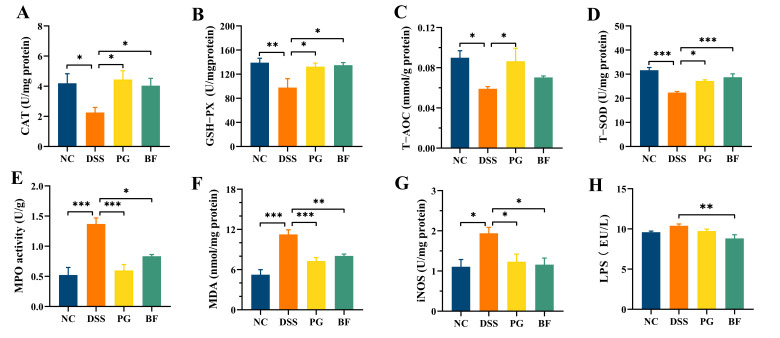
PG and BF alleviated oxidative stress in colon tissues, including (**A**) CAT, (**B**) GSH-PX, (**C**) T-AOC, (**D**) T-SOD, (**E**) MPO, (**F**) MDA, (**G**) LPS, and (**H**) iNOS. * *p* < 0.05, ** *p* < 0.01, and *** *p* < 0.001.

Oxidative stress is a hallmark of intestinal inflammation and is driven by excessive ROS production that overwhelms endogenous antioxidant defenses. At physiological levels, ROS act as signaling molecules that contribute to host defense; however, sustained overproduction disrupts redox homeostasis and damages cellular components [41]. In the DSS group, CAT, GSH-PX, T-AOC, and T-SOD levels were markedly decreased, whereas MPO, MDA, and iNOS were increased, indicating pronounced oxidative stress in colon tissues (Figure 4A–G). BF and PG markedly reversed these alterations, as evidenced by restoration of antioxidant capacity and reduction in oxidative damage, with no significant differences between the two strains. SOD catalyzes the dismutation of O_2_^−^ to H_2_O_2_, which is then decomposed by CAT [42]. GSH-PX further limits oxidative injury by reducing lipid hydroperoxides [43]. In contrast, MPO reacts with H_2_O_2_ to generate highly reactive complexes [44], and MDA is a well-established end product derived from lipid peroxidation, serving as a robust indicator of oxidative damage [45]. Overproduction of NO by iNOS may also contribute to cytotoxicity under inflammatory conditions [46]. Previous studies have shown that *P. goldsteinii* [47] and *B. fragilis* [48] alleviate colitis by mitigating oxidative damage. These results suggest that BF and PG alleviate colonic inflammation, at least in part, by restoring redox balance.

### 3.5. P. goldsteinii and B. finegoldii Preserved Intestinal Barrier Integrity

**Figure 5:**
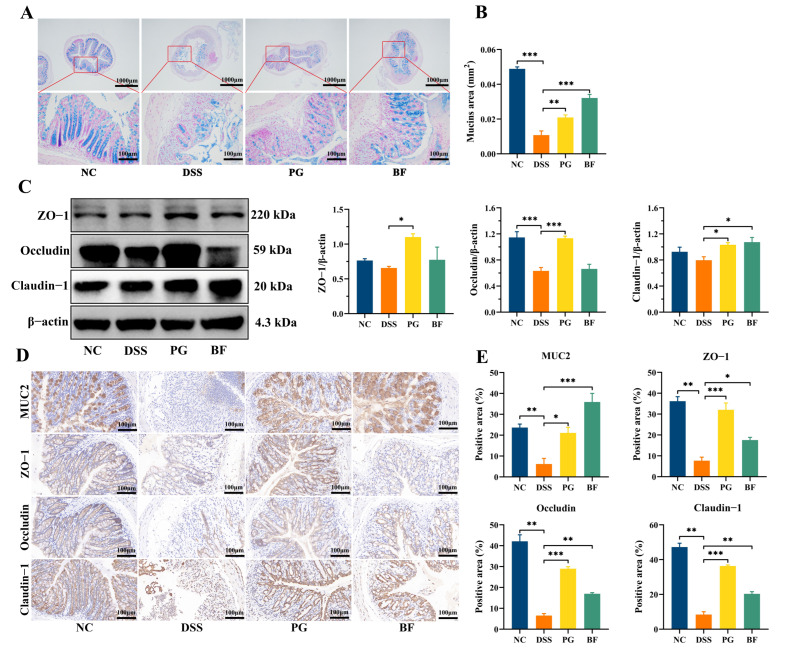
PG and BF maintained intestinal barrier integrity. (**A**) Representative AB-stained images of colon tissues (upper, scale bar = 1000 μm) and (lower, scale bar = 100 μm). (**B**) Quantification of mucin-positive area. (**C**) Western blot analysis and quantification of ZO-1, occludin, and claudin. (**D**) Immunohistochemical staining and (**E**) quantification analysis of MUC2, ZO-1, occludin, and claudin, scale bar = 100 μm. * *p* < 0.05, ** *p* < 0.01, and *** *p* < 0.001.

The intestinal barrier is a multilayered defense system that prevents the translocation of luminal antigens, toxins, and pathogens into host tissues. DSS-induced colitis markedly disrupted barrier structure, as reflected by crypt distortion, goblet cell loss, and mucosal thinning (Figure 5A,B). PG and BF alleviated these pathological changes and improved mucosal structure. At the molecular level, Western blot results showed that tight junction proteins ZO-1 (*p* > 0.05), claudin-1 (*p* > 0.05), and occludin were reduced in the DSS group (Figure 5C). PG treatment markedly restored their expression, whereas BF showed a partial effect, with a more evident improvement in claudin-1 and comparatively limited effects on ZO-1 and occludin. However, immunohistochemistry showed markedly reduced intensities of MUC2, ZO-1, occludin, and claudin-1 (*p* < 0.05) in DSS-treated mice, which was preserved to varying extents by PG and BF treatment (Figure 5D,E).

MUC2, secreted by goblet cells, forms a protective mucus layer that prevents bacterial contact with the epithelial cells [49]. Beneath this layer, tight junction complexes composed of claudin, occludin, and ZO-1 maintain epithelial cohesion and regulate paracellular permeability. Dysbiosis can compromise mucus availability and junctional integrity, thereby exacerbating tissue injury and immune activation [50]. A previous study showed that *Parabacteroides* and *Bacteroides* strains enhanced barrier function and reduced inflammation [51]. *P. distasonis* attenuated tumorigenesis in mice by reinforcing intestinal barrier [52], and *B. fragilis* ZY-312 conferred protection from radiation-induced injury via activation of the transcription 3 signaling pathway [53]. These results suggest that barrier preservation represents an important component of the protective effects of PG and BF on colitis.

### 3.6. P. goldsteinii and B. finegoldii Preserved Barrier Integrity by Suppressing MLCK Activation

**Figure 6:**
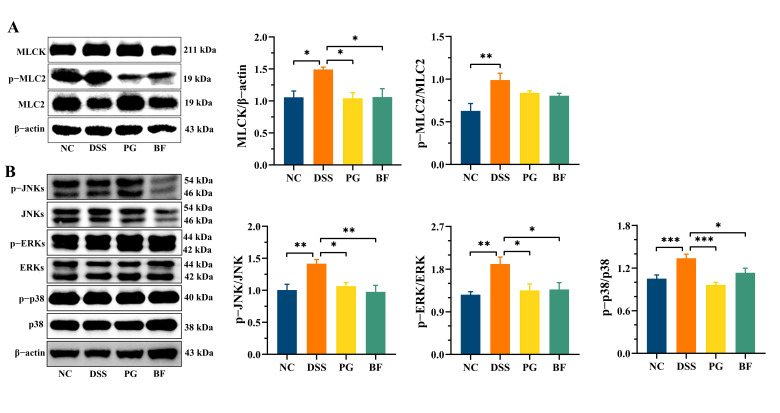
PG and BF suppressed activation of the MLCK/MAPK pathways in colon tissues. (**A**) Representative Western blot images and quantitation of MLCK expression and p-MLC2/MLC2 ratio. (**B**) Representative Western blot analysis and quantitation of MAPK pathway-related proteins. * *p* < 0.05, ** *p* < 0.01, and *** *p* < 0.001.

Epithelial barrier function relies on tight junction stability, which is dynamically regulated by the actin cytoskeleton. Myosin light chain kinase (MLCK) phosphorylates MLC2, promoting actomyosin contraction and facilitating tight junction disassembly, including destabilization of ZO-1. DSS treatment significantly increased MLCK expression and p-MLC2/MLC2 ratio, indicating activation of the MLCK/MLC pathway (Figure 6A). BF and PG treatment reduced MLCK expression and lowered p-MLC2/MLC2 ratio (*p* > 0.05), suggesting a trend toward inhibition of MLCK-mediated contractility. Inflammation can activate MLCK through the NF-κB and MAPK pathways. In this study, DSS exposure significantly increased p-JNKs/JNKs, p-ERKs/ERKs, and p-p38/p38 ratios (Figure 6B), indicating activation of the MAPK pathway. BF and PG significantly reduced these phosphorylation ratios, consistent with their suppression of pro-inflammatory cytokines. No marked difference was observed between the two strains. Notably, p38 has been implicated as a key mediator of inflammation-associated epithelial barrier disruption [54]. Previous studies showed that *Bacteroides* and *Parabacteroides* strains modulated this pathway to suppress *E. coli*-induced inflammation and preserve barrier integrity [55]. Similarly, *B. thetaiotaomicron* alleviated experimental colitis through p38 inhibition [56]. These results suggest that BF and PG reinforce epithelial integrity through regulation of MAPK signaling and downstream MLCK pathway.

### 3.7. P. goldsteinii and B. finegoldii Modulated Gut Microbiota Dysbiosis

**Figure 7:**
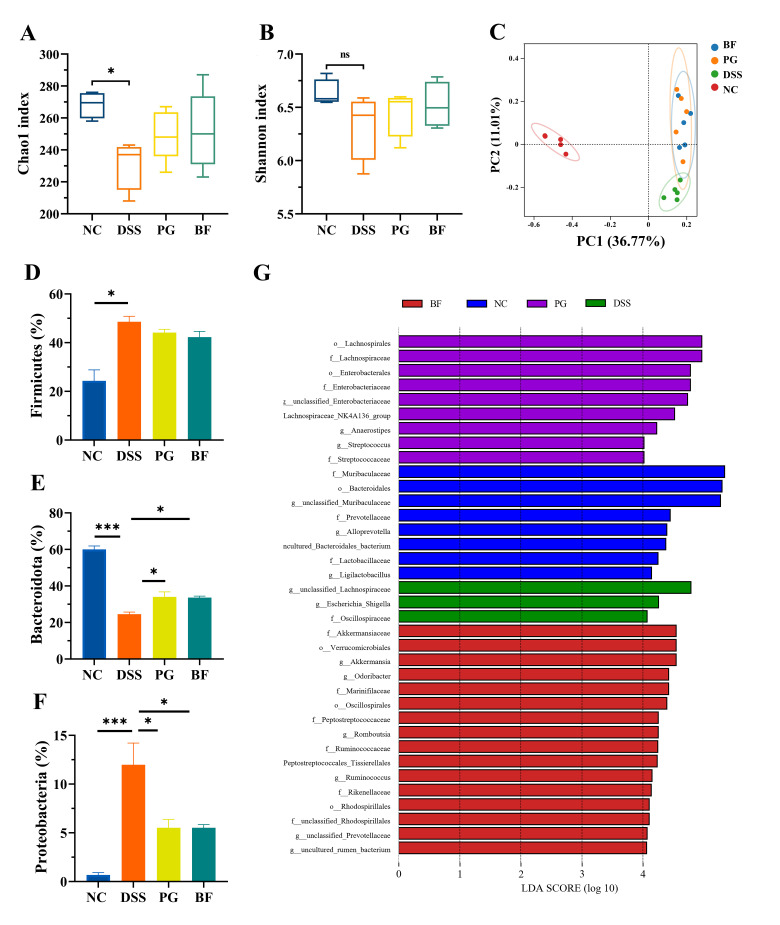
PG and BF modulated microbiota dysbiosis. (**A**) Chao1 index. (**B**) Shannon index. (**C**) PCoA based on Bray–Curtis distances. (**D**–**F**) Relative abundances of Firmicutes, Bacteroidota, and Proteobacteria. (**G**) LDA effect size (log 10 > 4). * *p* < 0.05 and *** *p* < 0.001. ns = not significant.

Gut microbiota dysbiosis and barrier dysfunction are hallmarks of intestinal inflammation. DSS treatment reduced microbial richness, as indicated by a decreased Chao1 index, whereas BF and PG partially restored richness (*p* > 0.05, Figure 7A). No marked changes were observed in the Shannon index among groups (Figure 7B). PCoA revealed a clear separation between the DSS and NC groups, while BF and PG shifted gut microbiota toward a unique, treatment-associated community structure (Figure 7C). No significant difference was detected between the BF and PG groups. These results are consistent with previous reports that *B. vulgatus* Bv46 and *P. goldsteinii* alleviated colitis via modulation of gut microbiota composition [13].

At the phylum level, the DSS group exhibited significant increases in Firmicutes and Proteobacteria, accompanied by a reduction in Bacteroidota. BF and PG decreased Proteobacteria and increased Bacteroidota without markedly affecting Firmicutes (Figure 7D–F). Proteobacteria overgrowth is a common feature in IBD and is positively correlated with disease severity [57]. Proteobacteria level is influenced by Firmicutes and Bacteroidota that constitute the majority of gut microbiota. Bacteroidota members, particularly *Bacteroides* and *Parabacteroides*, are often reduced during colitis and show an inverse association with disease severity [58].

LDA identified taxa specifically enriched in each group (Figure 7G). Nine taxa were enriched in the PG group, including Lachnospiraceae, Enterobacteriaceae, and Streptococcaceae, whereas 17 taxa were enriched in the BF group, including Akkermansiaceae, Peptostreptococcaceae, Marinifilaceae, Rikenellaceae, and Ruminococcaceae. Many of these taxa are renowned for short-chain fatty acid (SCFA) production, ROS neutralization, and maintenance of mucosal integrity. For instance, Ruminococcaceae and Rikenellaceae produce SCFAs that suppress TNF-α secretion and NF-κB activation [59], while Lachnospiraceae generate butyrate, an energy source for colonocytes and a promoter of regulatory T cell differentiation [60]. Akkermansiaceae has been shown to enhance tight junction protein expression and strengthen barrier function [13], and Rikenellaceae may contribute to reduced oxidative stress by neutralizing ROS [61]. These results suggest that BF and PG alleviate colitis by reshaping the gut microbiota toward a composition associated with barrier protection and reduced inflammation.

### 3.8. P. goldsteinii and B. finegoldii Reprogram Microbiota-Associated Metabolomic Profiles

**Figure 8:**
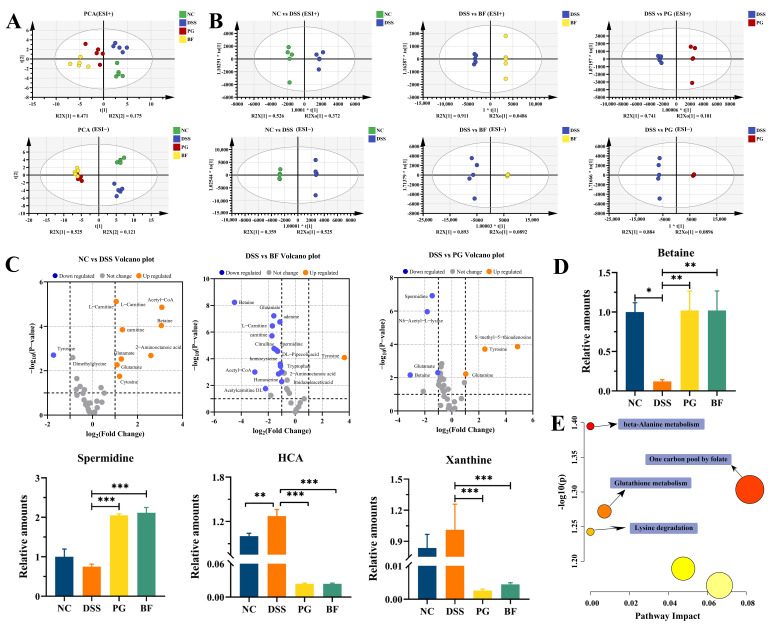
PG and BF ameliorated microbiota-associated metabolite abnormalities in colitis mice. (**A**) PCA based on ESI− and ESI+ metabolomic datasets. (**B**) OPLS-DA score plot based on ESI− and ESI+ modes. (**C**) Volcano plots showing differential metabolites for NC vs. DSS, DSS vs. BF, and DSS vs. PG comparisons. (**D**) Levels of key microbiota-associated metabolites. (**E**) Metabolic pathways analysis based on the KEGG database. * *p* < 0.05, ** *p* < 0.01, and *** *p* < 0.001.

Microbiota-derived metabolites play critical roles in maintaining host metabolic and immune homeostasis, and dysregulation of these metabolites is recognized as a hallmark of various diseases [62]. To assess the impact of PG and BF on metabolite profiles, metabolomic analysis was performed using positive (ESI+) and negative (ESI−) ion modes [63]. PCA and OPLS-DA revealed clear separation between the NC and DSS groups, while BF and PG partially restored the metabolite profiles (Figure 8A,B). Notably, the BF and PG groups also exhibited metabolomic signatures that were distinct from the NC group, implying strain-associated metabolic reprogramming. Similar metabolite shifts accompanying colitis alleviation have been reported for *B. thetaiotaomicron* and *P. distasonis* ATCC8503 [64,65].

Differential metabolites regulated by BF and PG were identified using volcano plots (Figure 8C) and displayed in Figure 8D. Bet was markedly reduced in the DSS group, whereas hydroxycarboxylic acid (HCA) and xanthine were slightly increased (*p* > 0.05). No obvious difference was observed in Spd between the DSS and NC groups (*p* > 0.05). BF and PG markedly increased Bet and Spd while reducing HCA and xanthine. Bet has been shown to enhance barrier function and suppress inflammatory responses [66], while Spd exerts anti-inflammatory effects by promoting macrophage M2 polarization and correcting gut dysbiosis [67]. Xanthine can contribute to oxidative stress and inflammatory signaling through xanthine oxidase activity [68]. Although the precise role of HCA in colitis remains unclear, it has been implicated in innate immune modulation. KEGG enrichment analysis showed that BF and PG significantly altered pathways related to glutathione metabolism and amino acid metabolism (Figure 8E). These pathways are known to support antioxidant defenses, epithelial integrity, and immune regulation during colitis. These results suggest that reprogramming of microbiota-associated amino acid and glutathione metabolism can contribute to the protective effects of BF and PG against colitis.

### 3.9. Spermidine and Betaine Alleviated Inflammation by Modulating Macrophage Polarization

**Figure 9:**
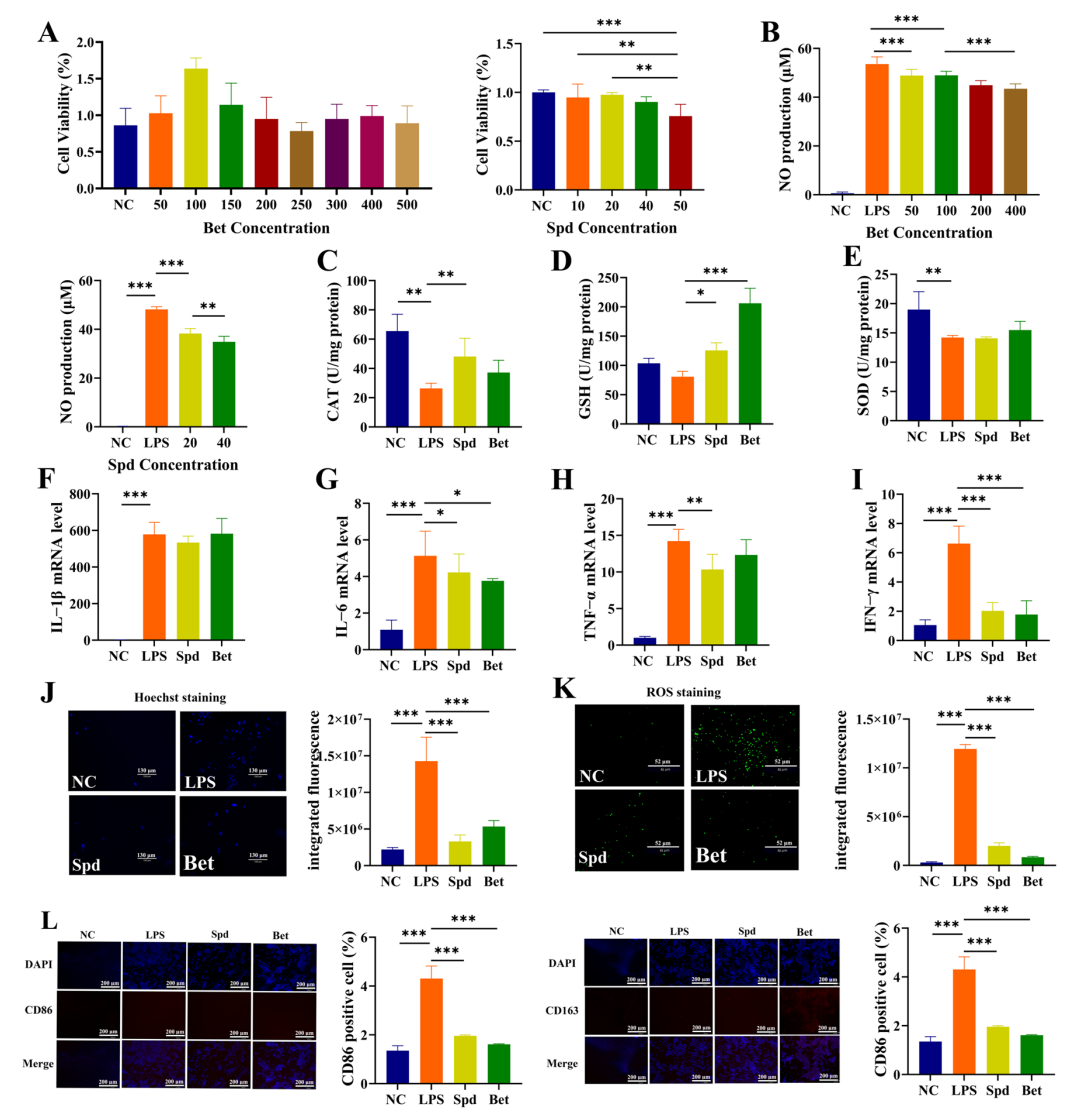
Bet and Spd mitigated LPS-stimulated inflammatory responses in RAW264.7 macrophages. (**A**,**B**) Effects of Bet and Spd on cell viability and NO production. (**C**–**E**) Levels of CAT, GSH, and SOD. (**F**–**I**) The levels of IL-1β, IFN-γ, TNF-α, and IL-6. (**J**) Apoptosis analysis based on Hoechst staining. (**K**) Intracellular ROS levels assessed by immunofluorescence staining. (**L**) Effects of Bet and Spd on macrophage polarization based on immunofluorescence staining of CD86 and CD163 and quantitative analysis. * *p* < 0.05, ** *p* < 0.01, and *** *p* < 0.001.

To investigate the roles of Spd and Bet in inflammation regulation, an LPS-stimulated RAW264.7 macrophage model was employed. MTT assays showed that Bet exhibited no cytotoxicity up to 500 μmol/L and Spd up to 40 μmol/L (Figure 9A). Bet (50–400 μmol/L) and Spd (20–40 μmol/L) dose-dependently reduced NO production (Figure 9B). Spd (50 μmol/L) markedly increased CAT activity and GSH level, whereas Bet (200 μmol/L) significantly increased GSH, with minimal effects on SOD activity and IL-1β level (Figure 9C–F). Spd significantly decreased the expression of IL-6, TNF-α, and IFN-γ, while Bet reduced IL-6 and IFN-γ levels (Figure 9G–I). Both metabolites also mitigated LPS-induced apoptosis and intracellular ROS accumulation (Figure 9J,K). Immunofluorescence analysis showed that Bet and Spd reduced CD86 (M1 marker) and increased CD163 (M2 marker), indicating a shift toward an anti-inflammatory macrophage phenotype (Figure 9L).

Macrophages play a central role in IBD pathogenesis. Pro-inflammatory M1 macrophages, induced by stimuli such as LPS and IFN-γ, amplify inflammation through cytokine release and ROS production, whereas M2 macrophages promote immune resolution and mucosal healing [69,70]. Amelioration of colitis is often associated with reduced M1 polarization and increased M2 responses [71]. M1 macrophages can enhance ROS production via the NF-κB and MAPK pathways, leading to apoptosis of tissue cells [71]. In this study, Spd and Bet promoted M2 polarization while suppressing M1-associated markers and pro-inflammatory cytokines, suggesting restoration of M1/M2 balance. These results are consistent with previous studies that Spd attenuated colitis by promoting M2 polarization [67] and that Bet favored anti-inflammatory macrophage responses through modulation of the TLR4/NF-κB pathway [72]. Notably, Spd and Bet have been shown to confer health benefits in humans, including modulation of autophagy and anti-inflammatory activities [73,74].

## 4. Conclusions

*Parabacteroides* and *Bacteroides* were identified as key mediators of fucoidan-induced attenuation of intestinal inflammation. BF and PG preserved epithelial barrier integrity, in part by suppressing MLCK activation, and reduced inflammatory responses by inhibiting the NF-κB/MAPK pathways. These protective effects correlated with microbiota remodeling and metabolite reprogramming, particularly increasing Spd and Bet. In vitro results showed that these metabolites alleviated LPS-induced inflammation by promoting anti-inflammatory macrophage polarization. These results highlight fucoidan as a promising functional food ingredient and support the potential of *Parabacteroides*/*Bacteroides* as next-generation probiotics. Our results provide a rationale for developing synbiotic strategies combining fucoidan with *Parabacteroides* and *Bacteroides*. Future studies should clarify the origins of key metabolites and validate their causality and translational relevance using approaches such as fecal microbiota transplantation, metabolite tracing, and human-relevant systems.

## Data Availability

The original contributions presented in the study are included in the article/Supplementary Materials. Further inquiries can be directed to the corresponding author.

## Supplementary Materials

The following supporting information can be downloaded at: https://www.mdpi.com/article/10.3390/foods15020203/s1, Table S1: Primer sequences used for RT-PCR.
