# Supplementary material for: Bacteroides finegoldii and Parabacteroides goldsteinii Mediate Fucoidan-Induced Attenuation of Intestinal Inflammation in Mice Through Betaine- and Spermidine-Related Pathways
Source: Foods. 2026 Jan 7;15(2):203. doi: 10.3390/foods15020203 (PMC12839593; doi:10.3390/foods15020203)
Supplement: Supplementary file 1 [file foods-15-00203-s001.zip › Table s1.pdf]

**Table S1** Primer sequences used for RT-PCR.

| Gene | Forward primer | Reverse primer |
|---|---|---|
| TNF-α | CGTGCTCCTCACCCACAC | GGGTTCATACCAGGGTTTGA |
| IL-1β | TCAGGCAGGCAGTATCACTCATT | GGAAGGTCCACGGGAAAGA |
| IL-6 | CCACTTCACAAGTCGGAGGCTTA | CCAGTTTGGTAGCATCCATCATTTC |
| INF-γ | GCCGATGATCTCTCTCAAGTGAT | ACAGCAAGGCGAAAAAGGATG |
